# Prolonged mechanical ventilation in critically ill patients: epidemiology, outcomes and modelling the potential cost consequences of establishing a regional weaning unit

**DOI:** 10.1186/cc10117

**Published:** 2011-03-27

**Authors:** Nazir I Lone, Timothy S Walsh

**Affiliations:** 1 Centre for Population Health Sciences, University of Edinburgh, Old Medical School, Teviot Place, Edinburgh, EH8 9AG, UK; 2 Department of Anaesthesia, Critical Care and Pain Medicine, Royal Infirmary of Edinburgh, Little France Crescent, Edinburgh, EH16 4SA, UK

## Abstract

**Introduction:**

The number of patients requiring prolonged mechanical ventilation (PMV) is likely to increase. Transferring patients to specialised weaning units may improve outcomes and reduce costs. The aim of this study was to establish the incidence and outcomes of PMV in a UK administrative health care region without a dedicated weaning unit, and model the potential impact of establishing a dedicated weaning unit.

**Methods:**

A retrospective cohort study was undertaken using a database of admissions to three intensive care units (ICU) in a UK region from 2002 to 2006. Using a 21 day cut-off to define PMV, incidence was calculated using all ICU admissions and ventilated ICU admissions as denominators. Outcomes for the PMV cohort (mortality and hospital resource use) were compared with the non-PMV cohort. Length of ICU stay beyond 21 days was used to model the effect of establishing a weaning unit in terms of unit occupancy rates, admission refusal rates, and healthcare costs.

**Results:**

Out of 8290 ICU admission episodes, 7848 were included in the analysis. Mechanical ventilation was required during 5552 admission episodes, of which 349 required PMV. The incidence of PMV was 4.4 per 100 ICU admissions, and 6.3 per 100 ventilated ICU admissions. PMV patients used 29.1% of all general ICU bed days, spent longer in hospital after ICU discharge than non-PMV patients (median 17 vs 7 days, *P *< 0.001) and had higher hospital mortality (40.3% vs 33.8%, *P *= 0.02). For the region, in which about 70 PMV patients were treated each year, a weaning unit with a capacity of three beds appeared most cost efficient, resulting in an occupancy rate of 73%, admission refusal rate at 21 days of 36%, and potential cost saving of £344,000 (€418,000) using UK healthcare tariffs.

**Conclusions:**

One in every sixteen ventilated patients requires PMV in our region and this group use a substantial amount of health care resource. Establishing a weaning unit would potentially reduce acute bed occupancy by 8-10% and could reduce overall treatment costs. Restructuring the current configuration of critical care services to introduce weaning units should be considered if the expected increase in PMV incidence occurs.

## Introduction

The requirement for a period of mechanical ventilation (MV) usually mandates admission to an ICU [1]. Most patients require short periods of respiratory support, but a minority require prolonged mechanical ventilation (PMV), which has been defined as a period of 21 days or more [2]. This definition is particularly relevant in the US health system because, due to financial drivers primarily, such patients are often subsequently transferred to long-term acute care (LTAC) facilities or specialised weaning units. The number of patients requiring MV is predicted to increase, in particular those who are elderly or with comorbidities, leading to a likely increase in the incidence of PMV [3]. Trends in the numbers of patients requiring PMV are of interest to health service planners because they consume a disproportionate amount of health care resources, and have high illness costs [4,5]. A recent study using the Medicare database showed a dramatic increase in the number of patients admitted to LTAC facilities in the US between 1997 and 2006, many of whom will fulfil the criteria for PMV [6]. The limited available data on patient outcomes indicate considerable variations between the populations studied [7].

In some countries, specialised weaning units have been established to manage patients who are stable except for the requirement for PMV. Potential advantages of these units include an increased focus on patient-centred rehabilitation, a greater concentration of management expertise, and improved patient outcomes [8]. Weaning units are potentially cost-effective because they require lower staff-to-patient ratios than ICUs [9]. Few weaning units exist in the UK, and LTAC facilities are not part of the health care model. As a result, most patients requiring long-term critical care remain in acute hospitals under the care of intensivists until successfully weaned from MV [9]. The UK system is therefore well suited to model the potential impact of weaning units to manage PMV cases.

The aim of this study was to use routinely collected audit data to establish the incidence of PMV in a health care administrative region of the UK, to report characteristics and outcomes of the PMV cohort, and to model the potential impact of establishing a weaning unit on costs and services.

## Materials and methods

### Design

We performed a retrospective cohort study using prospectively collected data available in the Scottish Intensive Care Society Audit Group (SICSAG) database [10]. The Local Research Ethics Committee (Lothian Research Ethics Committee 3) waived the need for formal ethical review. Patient confidentiality was ensured as the dataset was fully anonymised.

### Setting

Our study was based in Lothian Health Board, which serves a population of 900,000 in South East Scotland. The region is served by three adult hospitals managed as a single organisation providing general ICU services as well as some national and specialist regional services: neuroscience (including traumatic brain injury), solid organ transplantation (excluding heart/lung), trauma, acute liver failure, and complex vascular surgery. Each hospital has a "closed" adult general mixed medical/surgical ICU with specialist intensive care staff. The number of funded ICU beds in the region varied from 23 to 26 over the study period [see Additional file 1, Table S1]. In total, the three ICUs typically manage about 1,100 MV patients per year. Referral patterns for South East Scotland mean that ICU patients comprise almost all patients requiring critical care in the geographical region, as well as some patients from outside the region for whom national or wider regional specialist services are provided. Cardiac surgery and coronary care services are managed in separate units and are not included in this analysis. Paediatric services are also not considered.

### Database

Every patient admission episode to an adult general ICU in Scotland generates a record that is stored in the SICSAG database [10]. A national validation study showed that the data quality was of a high standard when compared with clinical case notes, with only a 6% level of disagreement [11]. All data are entered prospectively by clinical staff and include demographics, Acute Physiology and Chronic Health Evaluation (APACHE) II scoring, and diagnostic data. Organ support data (e.g. invasive ventilation, renal replacement therapy (RRT), vasoactive therapy) and presence of a tracheostomy are entered on a daily basis during an admission episode. RRT in the three units is usually continuous haemofiltration, although intermittent haemodialysis is occasionally provided for more stable patients. Non-invasive ventilation is available in all units, but is not recorded in the SICSAG database. Survival status at ICU and hospital discharge is also recorded.

### Patient cohorts

All patient records were extracted from the SICSAG database from the three ICUs over a five-year period, from 1 January 2002 to 31 December 2006. The following two groups were excluded prior to analysis: patients under 16 years old and patients transferred between ICUs with incomplete data for at least one admission episode. These incomplete admission episodes resulted from patient transfers to/from ICUs that did not contribute to the Lothian regional dataset.

### Variables

Comorbidities were obtained from APACHE II chronic health evaluation categories. Admission diagnosis was derived from APACHE III diagnostic categories, with new groupings created by manual review by one of the authors (NL) for pneumonia, trauma, and sepsis [see Additional file 1, Table S2]. Outcome variables were recorded in the SICSAG dataset. No assumptions were made regarding missing data, and analyses were undertaken using a complete-case analysis.

### Calculation of PMV incidence

Calculation of incidence requires an agreed numerator and denominator, and neither are universally agreed when describing the epidemiology of PMV. For numerator data, we used two definitions of PMV to provide sensitivity analyses given the potential limitations of retrospective data as detailed below.

#### Consecutive PMV (consecPMV)

An international consensus defined PMV as at least six hours of ventilation per day for 21 consecutive days [2]. In the SICSAG database, a ventilated day is defined as ventilation for any period of time during the preceding 24 hours. For admission episodes in 2005 and 2006, the number of consecutive days ventilated were available. For these two years the data enabled us to identify patients who required at least 21 consecutive days of MV (or endotracheal tube *in situ*). We allowed a period of discontinuation of ventilation of one day or less within this definition to allow for recording errors or brief MV free periods. This definition was termed consecutive PMV (consecPMV).

#### Counted PMV (countPMV)

The total number of ventilated days during an ICU stay, without reference to the number of consecutive ventilated days, was available for the entire dataset (2002 to 2006). To maximise information from the available dataset in analyses, a second measure of PMV was defined as ventilation for 21 days or more with no reference to consecutive days ventilated. This definition was termed counted PMV (countPMV).

For denominator data, we used two different measures. First, all ICU admissions irrespective of MV status during ICU stay (ICUtotal); second, all ICU admissions who required MV at any time during ICU stay (ICUvent). Trends in the annual incidence of PMV were calculated for the study period (2002 to 2006) using countPMV as the numerator, and both measures of denominator. In addition to these measures, we undertook a sensitivity analysis in which we included the group with incomplete admission episodes to recalculate incidence using countPMV as the numerator. We classified episodes in this group as PMV if the length of hospital stay before or after the index ICU admission made it possible that ventilation for 21 days or more occurred.

### Resource utilisation

The number of bed-days used by the PMV population was calculated as a proportion of total funded level three bed-days in the three Lothian ICUs over the study period. A level three bed is one in which a patient receives MV and/or multi-organ support. Annual estimates of number of funded ICU bed-days in Lothian were obtained from local service managers.

### Modelling a regional weaning unit

We limited modelling to data from the years 2005 and 2006 because consecutive daily organ support data were available for this subgroup. Four different hypothetical weaning units were modelled using different admission criteria (see Table 1). In all cases we used consecPMV as the numerator.

**Table 1:** Eligibility criteria for four models of weaning units

	Unit A	Unit B	Unit C	Unit D
Minimum period of consecutive ventilation	21 days	21 days	21 days	21 days
Minimum period free from RRT prior to transfer	7 days	2 days	RRT allowed	RRT allowed
Minimum period free from vasoactive treatment prior to transfer	7 days	2 days	7 days	2 days

RRT, renal replacement therapy.

Criteria were chosen after discussion with intensive care clinicians locally and a review of the literature, acknowledging that weaning units were unlikely to admit patients still requiring cardiovascular support, and may not be able to provide RRT. In general, unit A represented the most stable patients requiring only ventilatory support who were likely to have a low risk of clinical deterioration. In contrast unit D represented patients requiring ongoing renal support in whom cardiovascular support was only recently discontinued, and who may have been at greater risk of clinical deterioration. Units B and C were modelled to include patients at intermediate levels of risk.

For modelling, the analyses were undertaken using syntax programming in the statistical software package SPSS (SPSS Inc., Chicago, IL, USA), and the results were crosschecked manually for unit A in a spreadsheet package (not shown). The date of eligibility for each PMV patient for each of the four hypothetical units was determined, excluding patients who never reached eligibility criteria. In this eligible patient group, no patient had more than one admission episode. For each hypothetical unit, baseline characteristics of eligible patients were described, together with ultimate hospital outcome. We used the remaining length of stay in ICU after eligibility from individual level patient data to calculate occupancy rates, varying capacity from one to eight beds for each of the four weaning units. We also estimated the likely refusal rates for admission as a result of inadequate capacity by assessing weaning unit bed availability on the day that a patient became eligible for admission to the weaning unit. If the unit was full on this day, this was counted as a refusal. Costs were calculated using a top-down approach which included physician and staff costs. Prices were converted from UK£ to euros (€) using the Organisation for Economic Co-operation and Development Purchasing Power Parity rate of €1 = UK£0.822292 [12]. An ICU bed-day was assumed to cost €1,690 per day (£1,390 published UK National Health Service (NHS) tariff 2009/10) [13], and a weaning unit bed around half this value [9]. The reduced cost of weaning unit beds is due to the expected lower staffing ratios required. A sensitivity analysis was undertaken varying the cost of a weaning unit bed from 50% to 100% of the cost of an ICU bed. The model assumed that staffing an unoccupied bed in the weaning unit would accrue the same cost as an occupied bed. In addition, eligible patients would only be transferred once a bed became available.

### Statistical analysis

All analyses were undertaken using SPSS v14. Patient characteristics were presented as number and percentage, mean and standard deviation (SD), and/or median and interquartile range (IQR). Both mean and median values were reported for continuous variables with skewed distributions, which were important in reporting resource use, for example length of ICU stay. Characteristics were described for PMV and non-PMV groups, and compared with the following tests: t-test for normally distributed data, Mann-Whitney U test for non-normally distributed data, and chi-squared test for categorical variables. Trends were analysed using chi-squared test for trend for categorical variables. The association between PMV status and diagnostic category was assessed using relative risk, although only those with a *P *value less than 0.0004 were considered to be significant to correct for multiple comparisons. Confidence intervals (CI) for incidence rates were derived using the Poisson distribution [14]. A significance level of 5% was used for analyses and 95% CI were presented (unless stated).

## Results

**Figure 1:**
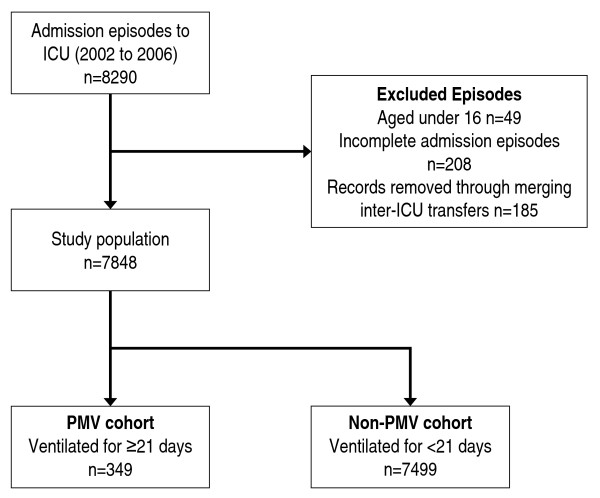
****Flow diagram indicating derivation of study population****. PMV, prolonged mechanical ventilation.

Between 2002 and 2006, there were 8,290 admission episodes to the three ICUs, of which 257 (3.1%) were excluded according to pre-defined criteria. Merging multiple episodes for the same patient reduced the number of patient episodes by 185, leaving 7,848 admission episodes, which constituted the study population (Figure 1). Baseline characteristics for patients with incomplete admission episodes (*n *= 208) had similar baseline characteristics to the study population although a higher proportion required PMV (11.1% vs 4.4%) [see Additional file 1, Table S3].

### Comparison of PMV and non-PMV groups

Patients who required PMV (defined as countPMV) when compared with non-PMV patients for the years 2002 to 2006 were older, more likely to be non-surgical admissions, had higher overall severity of illness on admission, and worse oxygenation on day one, but had fewer recorded comorbidities (Table 2). Pneumonia, septic shock, and trauma were among the most common five reasons for admission in both groups. However, only five ICU admission diagnostic categories were associated with a significantly higher risk of PMV (*P *< 0.0004 for all comparisons): Guillain-Barré syndrome (relative risk (RR) = 12.2), pancreatitis (RR = 4.6), acute respiratory distress syndrome (RR = 4.0), pneumonia (RR = 3.4), and septic shock (RR = 2.1).

**Table 2:** Baseline characteristics and outcomes of PMV and non-PMV cases in study population

	n*				
				
	PMV	Non-PMV	PMV	Non-PMV	*P *value
Age mean (SD)	349	7,499	59.6 (15.2)	56.9 (18.1)	0.001
Sex n (% female)	349	7,499	148 (42.4)	3,218 (42.9)	0.86
Days in hospital before ICU admission					<0.001*^a^*
Mean (SD)			5.9 (14.0)	3.5 (11.0)	
Median (IQR)			1 (0 to 5)	0 (0 to 2)	
APACHE II score mean (SD)	307	6,659	21.0 (6.8)	18.8 (8.3)	<0.001
CPR in 24 hours before ICU admission n (%)	349	7,499	23 (6.6)	663 (8.8)	0.15
Number of co-morbidities n (%)	340	7,228			<0.001*^a^*
None			276 (81.2)	5,317 (73.6)	
1			50 (14.7)	1,211 (16.8)	
2 or more			14 (4.1)	700 (9.7)	
Surgical status n (%)	347	7,463			<0.001
Emergency			52 (15.0)	1,747 (23.4)	
Elective			15 (4.3)	893 (12.0)	
Non-surgical			280 (80.7)	4,823 (64.6)	
Ventilated on day 1 n (%)	341	7,354	312 (91.5)	4,884 (66.4)	<0.001
PaO_2_:FiO_2 _ratio (mmHg)	334	6,477			<0.001
Mean (SD)			155 (82)	242 (125)	
Median (IQR)			139 (93 to 201)	227 (142 to 322)	
Tracheostomy placed during admission n (%)	349	7,499	219 (62.8)	470 (6.3)	<0.001
Five most common admission diagnoses n (%)	349	7,499			-
Pneumonia - any cause			92 (26.4)	650 (8.7)	
Septic shock - any source			39 (11.2)	398 (5.3)	
Trauma			22 (6.3)	466 (6.2)	
Gastrointestinal perforation			18 (5.2)	-	
Pancreatitis			18 (5.2)	-	
Cardiac arrest			-	457 (6.1)	
Self-inflicted overdose			-	401 (5.3)	
Length of ICU stay (days)	349	7,499			-
Mean (SD)			37.2 (16.1)	3.8 (4.9)	
Median (IQR)			33 (26 to 44)	2 (1 to 5)	
Number of days ventilated	349	7,499			-
Mean (SD)			33.2 (14.7)	2.9 (4.2)	
Median (IQR)			29 (24 to 38)	1 (0 to 3)	
Length of hospital stay after ICU discharge (days)	296	6,709			<0.001*^a^*
Mean (SD)			35 (49)	19 (40)	
Median (IQR)			17 (0 to 45)	7 (0 to 20)	
Hospital discharge destination of survivors n (%)	179	4,436			0.06
Normal residence			160 (89.4)	4,155 (93.7)	
Rehabilitation unit			14 (7.8)	224 (5.0)	
Long-term institutional care			5 (2.8)	49 (1.1)	
Hospice or equivalent			0 (0)	5 (0.1)	
ICU mortality n (%)	317	7,103	83 (26.2)	1,654 (23.3)	0.23
Hospital mortality n (%)	305	6,763	123 (40.3)	2,286 (33.8)	0.02

Readmission episodes are excluded (*n *= 428) for length of hospital stay, hospital discharge destination, ICU mortality and hospital mortality. *Due to missing data, n is stated for each variable. *^a^*Mann-Whitney U test.

APACHE, Acute Physiology and Chronic Health Evaluation; CPR, cardiopulmonary resuscitation; FiO2, fraction of inspired oxygen; IQR, interquartile range; PaO2, partial pressure of arterial oxygen in mmHg; PMV, prolonged mechanical ventilation; SD, standard deviation.

A comparison of outcomes for PMV and non-PMV groups is shown in Table 2. PMV patients had a non-significant increase in ICU mortality (absolute difference = 2.9%, 95% CI -1.9% to 8.3%, *P *= 0.23), which reached statistical significance at hospital discharge (absolute difference = 6.5%, 95% CI = 1.1% to 12.2%, *P *= 0.02). PMV patients had longer post-ICU hospital stays than non-PMV patients (*P *< 0.001). Patterns of discharge from the acute hospital were similar for the two groups (*P *= 0.06), with a high proportion of PMV patients discharged to their own residence from the acute hospital (89%) and small numbers being transferred to rehabilitation or long-term care facilities (11%).

### PMV incidence and resource use

The incidence of PMV calculated using different definitions of numerator and denominator is shown in Table 3. For countPMV (data 2002 to 2006) the incidence was 4.4 per 100 ICU admissions using all admissions as the denominator and 6.3 per 100 ventilated ICU admissions. For consecPMV (data 2005 to 2006) the incidence was 3.7 per 100 admissions, which compared with an incidence of 3.9 per 100 admissions when countPMV was calculated for the same period.

**Table 3:** Measuring incidence of PMV using different numerators and denominators

Time period	Numerator and denominator	n	Incidence (per 100 admissions)	95% CI
2002-2006	countPMV	349	4.4	4.0 to 4.9
	ICUtotal	7,848		

2002-2006	countPMV	349	6.3	5.7 to 7.0
	ICUvent	5,552		

2005-2006	consecPMV	126	3.7	3.1 to 4.4
	ICUtotal	3,442		

2005-2006	countPMV	135	3.9	3.3 to 4.6
	ICUtotal	3,442		

CI, confidence interval; ICUtotal, all ICU admissions; ICUvent, ICU admissions requiring mechanical ventilation; PMV, prolonged mechanical ventilation. For definitions of countPMV and consecPMV see text.

In the sensitivity analysis, inclusion of the group with incomplete admission episodes (*n *= 208) gave a countPMV incidence of up to 5.8 per 100 ICU admissions (95% CI = 5.3 to 6.3), or 8.8 per 100 ventilated ICU admissions (95% CI = 8.0 to 9.6).

Over the entire study period (2002 to 2006) there were, on average, 24.4 funded level three ICU beds per year in the region (Table 1), and there was a mean of 70 PMV cases annually. Mean ICU length of stay per PMV case was 37.2 days, equating to 29.1% of all funded level three ICU bed days over the study period.

### Modelling a regional weaning unit

Out of 126 patients requiring PMV during the period 2005 to 2006, between 80% (unit A) and 93% (unit D) were eligible for transfer to the hypothetical weaning units. Table 4 summarises the eligibility criteria, patient characteristics, outcomes, and bed-day use of the cohorts eligible to be admitted to each of the four weaning units. Mortality was lower at ICU discharge and hospital discharge in unit A, the unit which accepted more stable patients (no RRT or vasoactive support for seven days prior to transfer).

**Table 4:** Characteristics and outcomes of PMV patients eligible for four different models of weaning units

	Unit A (*n *= 101)	Unit B (*n *= 113)	Unit C (*n *= 110)	Unit D (*n *= 117)
				
Weaning unit description	No vasoactive treatment or RRT for 7 days	No vasoactive treatment or RRT for 2 days	No vasoactive treatment for 7 days	No vasoactive treatment for 2 days
Age (mean (SD))	59 (15)	58 (16)	59 (16)	58 (16)
Sex n (%) female	45 (45)	48 (43)	48 (44)	50 (43)
Surgical status n (%)				
Elective	2 (2)	2 (2)	2 (2)	2 (2)
Emergency	11 (11)	12 (11)	13 (12)	13 (11)
Non-surgical	88 (87)	99 (88)	95 (86)	102 (87)
APACHE II score (mean (SD))*^a^*	20.7 (7.0)	20.7 (7.0)	21.0 (6.9)	20.9 (7.0)
Tracheostomy on reaching eligibility for unit; n (%)	94 (93)	102 (90)	102 (93)	106 (91)
Length of ICU stay after reaching eligibility (days)				
Mean (SD)	14.9 (13.4)	14.9 (13.9)	14.9 (13.5)	15.3 (14.0)
Median (IQR)	11 (6 to 22)	11 (5 to 22)	11 (5 to 22)	11 (5 to 23)
Days ventilated after reaching eligibility				
Mean (SD)	11.3 (11.8)	11.6 (12.2)	11.5 (11.9)	12.0 (12.2)
Median (IQR)	7 (3 to 17)	7 (3 to 17)	7 (3 to 17)	8 (4 to 17)
Proportion of funded ICU bed-days used by population after reaching eligibility	8.1%	9.1%	8.9%	9.7%
Possible readmissions to ICU per year n (%)*^b^*	15 (15)	21 (19)	17 (16)	21 (18)
Mortality at ICU discharge n (%)	14 (14)	19 (17)	19 (17)	22 (19)
Mortality at final hospital discharge n (%)*^c^*	29 (32)	35 (34)	34 (34)	38 (36)

^*a *^n for APACHE II score: *n *= 88, *n *= 97, *n *= 96, *n *= 101; ^*b *^patients who would be transferred out of the weaning unit due to clinical instability, either requiring vasoactive therapy or RRT (units A and B) or vasoactive therapy alone (units C and D); ^*c *^n for mortality at final hospital discharge: *n *= 91, *n *= 102, *n *= 99, *n *= 106 for units A to D, respectively.

APACHE, Acute Physiology and Chronic Health Evaluation; IQR, interquartile range; PMV, prolonged mechanical ventilation; RRT, renal replacement therapy; SD, standard deviation.

**Figure 2:**
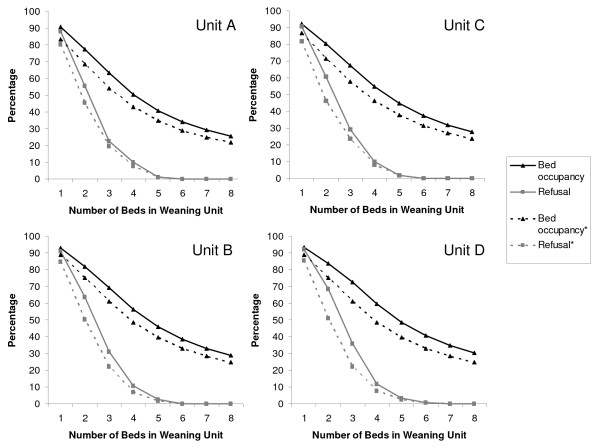
**Modelling bed occupancy and admission refusal rates for four weaning units (A to D) with differing bed capacities**. A refused admission occurs when a patient is eligible for transfer from ICU to the weaning unit, but the weaning unit is full. *The broken lines represent the effect of unstable patients being transferred out of the weaning unit back to the ICU.

The proportion of funded ICU bed-days used by these cohorts of patients varied from 8.1% to 9.7%, equivalent to a mean of 2.0 to 2.4 ICU beds occupied per day in the region over the two-year period. Figure 2 demonstrates how occupancy and refusal rates changed for weaning units A to D when capacity was varied from one to eight beds. For example, a three-bed unit with eligibility criteria for unit D would have a bed occupancy rate of 72.6%. However, a new admission would be refused admission on the day eligible for transfer to a weaning unit on 36% of occasions. A five-bed unit for unit D would have a bed occupancy of 48.6% and refusal rate for new admissions of 3%.

To take account of the effect of patients becoming unstable once transferred to a weaning unit, the modelling exercise was repeated. In this model, those patients who deteriorated and required cardiovascular support in units C and D, or renal and cardiovascular support in models A and B, were assumed to have been transferred out of the weaning unit and readmitted to the ICU on the first day of requiring organ support. For reasons of simplicity, once a patient was readmitted to the ICU, they were no longer eligible for transfer back to the weaning unit. The effect of this reduction in weaning unit bed occupancy and refusal rates is represented in Figure 2 using broken lines.

**Figure 3:**
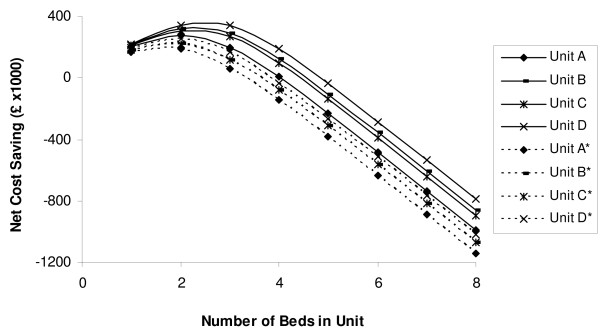
**Net cost saving of establishing a weaning unit with differing bed capacities**. *The broken lines represent the effect of unstable patients being transferred out of the weaning unit back to the ICU.

The potential cost saving gained from transferring patients to a weaning unit was modelled accounting for a limited bed capacity by varying unit capacity from one to eight beds (Figure 3). For unit D, a three-bed unit offers the highest cost saving of around £344,000 (€418,000) per year. Staffing a five-bed unit would no longer create a net saving. Instead, there would be a net cost of £35,000 (€43,000) per year. Once unstable patients who are transferred back to the ICU are taken into consideration, establishing unit D with four beds no longer has an associated cost saving (net cost £32,000 (€39,000) per year; Figure 3 broken line). The results of the sensitivity analysis, which was undertaken by varying the cost of a weaning unit bed from 50% to 100% of the cost of an ICU bed, are available in the Additional file 1 (Figures S1 and S2). It showed that once a weaning unit bed reached 70% of the cost of an ICU bed, a three-bed unit would no longer yield a cost saving for unit A.

## Discussion

We found the incidence of PMV was 4.4 per 100 ICU admissions or 6.3 per 100 ventilated admissions in a UK health care administrative region serving a population of 900,000. These patients spent a mean of 37 days in the ICU and utilised 29% of all ICU bed days. When we modelled the potential impact of establishing a regional weaning unit we found that 8 to 10% of ICU beds might be vacated, and that there was potential for considerable cost savings (£344,000 (€418,000) per annum in the best scenario).

### Strengths and limitations

The major strength of our study was the inclusion of 95% of all ICU admissions in the region over the study period, which minimised selection bias. Other studies have described a subpopulation of PMV patients following transfer to a weaning unit [15], or were unable to report characteristics of excluded patients [4]. We were also able to undertake a range of sensitivity analyses based on different definitions and inclusion of cohorts with uncertain PMV status. These analyses did not show clinically important variations in our estimates of incidence, suggesting they were accurate. Two groups of patients cared for in specific clinical areas were excluded from our study cohort because these areas did not submit data to the SICSAG database. Although most patients with neurological injury who required PMV were included in the study cohort, a small number who received ongoing invasive ventilatory support in the neurosurgical high dependency unit had this period of ventilation excluded. In addition, any patients ventilated in cardiothoracic ICU were excluded. Studies reporting PMV rates following cardiac surgery use a shorter period of ventilation to define PMV, making a comparison difficult with our findings. Two UK studies reported PMV rates of 5.5% (ventilation >48 hours) [16] and 2.6% (ventilation >96 hours) [17] in these patients. Therefore, although the patients are few in number, this could have resulted in a small underestimation of the overall regional incidence rate.

There are a number of limitations to our modelling exercise. The retrospective nature of our study meant that data relating to ventilatory modes, sedation practice, and weaning methods were not available. Advances in clinical practice, such as daily sedation breaks [18], spontaneous breathing trials [19], and weaning protocols [20] may help to reduce the length of MV and potentially reduce the need for establishing a weaning unit. We did not take the potential impact of a weaning unit on outcomes into account, for example, reduced duration of MV or improved mortality. A reduction in MV duration would further improve net cost savings and decrease patient refusals. In addition, we assumed patients would only be transferred after 21 days, whereas in practice transfer might occur at an earlier stage if appropriate, and according to bed pressures. These factors are difficult to adjust for in a modelling exercise, but in general would probably improve the efficiency of resource use. The method of costing health care in the UK is difficult to generalise to other health care systems, in part because costs are allocated using a 'top-down' approach rather than a per item 'bottom-up' approach [21]. Our estimates of cost included the assumption that both occupied and unoccupied weaning unit beds incurred similar costs, and we also did not consider the potential for transfer of suitable patients prior to 21 days of MV. These factors mean we have probably underestimated potential cost savings, although we did not include the capital costs associated with setting up a weaning unit. Despite this, our models are the first to use high-quality clinical data and consider different entry criteria, including adjustment for clinical deterioration. We also evaluated bed occupancy and patient refusal rates, which has not previously been undertaken.

### PMV epidemiology

Incidence estimates are influenced strongly by the denominator utilised, especially for critical care populations for which case-mix is dependent on health care organisation. The most externally valid denominator is probably patients who require MV in the ICU. Our data suggest a PMV incidence of 6.3 per 100 ventilated ICU patients in a typical UK health board region (or equivalent) for this population. Few studies of PMV incidence have been published and variations in both denominator and numerator definitions make direct comparison with our study difficult. Using the consensus definition of 21 days or longer of MV, a single-centre Argentinean study reported a PMV incidence of 14.3 per 100 ICU admissions [22], which is considerably greater than our cohort. Using a PMV definition of 21 days or longer of MV and a denominator of patients ventilated for 48 hours or longer, a prospective study based in a single US centre found that 14.0 per 100 patients required PMV [23]. This compares with a rate of 9.6 per 100 patients using similar definitions for numerator and denominator in our cohort (data not shown). A multicentre population based study in the US reported an incidence of PMV of 7.7 per 100 ventilated ICU admissions [24]. However, the numerator was defined as four days or longer ventilated with a tracheostomy and the denominator excluded elective surgical patients ventilated for less than 24 hours. Only one other UK study has reported PMV frequency. The incidence of stable PMV (≥14 days ventilated) patients who fulfilled criteria for a weaning unit was 2.5 per 100 ICU admissions [4]. Our data provide a number of measures of population incidence to improve generalisability of the findings.

The overall ICU and hospital mortality rates for the PMV cohort of 26% and 40% are consistent with severe critical illness. The higher death rate in the PMV cohort compared with the non-PMV cohort is likely related to the ongoing burden of chronic critical illness experienced by these patients [23]. Despite this, we found that 83% of PMV patients who were discharged alive from the ICU survived to hospital discharge. Direct comparison with published outcomes from other studies is difficult because most published cohorts had different entry criteria or were selected by admission to a weaning unit [25-29].

A high proportion of patients were discharged to their own residence from the acute hospital, suggesting that rehabilitation is occurring primarily in the acute environment and/or that patients are discharged with significant ongoing rehabilitation requirements. A recent UK guideline has highlighted the need to improve rehabilitation following critical illness [13]. Establishing dedicated rehabilitation facilities may have the benefit of reducing hospital stay and improving patient outcomes.

### Weaning unit modelling

Our modelling suggested that a three- or four-bed weaning unit was the most cost-efficient for our region. In practice it is likely that admission criteria might include patients from each of the four scenarios, with a key practical consideration being whether RRT in the form of intermittent haemodialysis would be provided in the unit. A geographically closer weaning unit would make provision of RRT or readmission to the ICU in the event of clinical deterioration more feasible. Only one other study has modelled a weaning unit in the UK. Robson et al. [4] used 14 days of ventilation rather than 21 days as the criterion for eligibility and required seven days of clinical stability prior to transfer. They found that 6.0% of ICU beds in their region were occupied by patients eligible for their weaning unit. Despite the limitations of modelling, our data suggest that a weaning unit could reduce the cost of providing critical care in our region if the equivalent number of acute ICU beds were reduced.

Further research is needed to evaluate the effect of establishing a weaning unit on patient-centred outcomes along with a more detailed economic evaluation. To date, published reports from US centres support possible improvements in some outcomes [30,31], and a reduction in costs [31].

## Conclusions

One in every 16 ventilated patients requires PMV in our region and this group use a substantial amount of health care resources. Establishing a weaning unit would potentially reduce acute bed occupancy by 8 to 10% and could reduce overall treatment costs. Restructuring the current configuration of critical care services to introduce weaning units should be considered if the expected increase in PMV incidence occurs.

## Key messages

• The incidence of PMV in a health care region of the UK is 4.4 per 100 ICU admissions and 6.3 per 100 ventilated ICU admissions.

• Patients requiring PMV utilise almost one-third of all ICU bed days available.

• Establishing a weaning facility on a regional basis could reduce acute ICU bed occupancy by 10% and reduce overall health care costs associated with treating critically ill patients.

## Abbreviations

APACHE: Acute Physiology and Chronic Health Evaluation; CI: confidence interval; consecPMV: consecutive PMV; countPMV: counted PMV; ICUtotal: all ICU admissions; ICUvent: ICU admissions requiring mechanical ventilation; IQR: interquartile range; LTAC: long-term acute care facility; MV: mechanical ventilation; NHS: UK National Health Service; PMV: prolonged mechanical ventilation; RR: relative risk; RRT: renal replacement therapy; SD: standard deviation; SICSAG: Scottish Intensive Care Audit Group.

## Competing interests

NL has received educational grants (unrelated to this work) from Eli-Lilly to attend educational events and critical care conferences. TSW has received unrestricted educational grants (unrelated to this work) from GE Healthcare, Wyeth Pharmaceuticals, and OthoBiotech.

## Authors' contributions

Both authors participated in the conception and design of the study. NL performed the statistical analysis. Both authors contributed to the manuscript draft, and both authors read and approved the final manuscript.

## Supplementary Material

Additional file 1 **Supplementary tables and figures**. Table S1: Number of funded ICU beds over the five-year study period. Table S2: Derivation of new diagnostic groupings from Acute Physiology and Chronic Health Evaluation (APACHE) III diagnostic codes. Table S3: Baseline characteristics and outcomes of study population and excluded group. Figure S1: Sensitivity analysis of cost saving of establishing a weaning unit for unit A. Figure S2: Sensitivity analysis of cost saving of establishing a weaning unit for unit D. Click here for file
