# Supplementary material for: Prolonged mechanical ventilation in critically ill patients: epidemiology, outcomes and modelling the potential cost consequences of establishing a regional weaning unit
Source: Crit Care. 2011 Mar 27;15(2):R102. doi: 10.1186/cc10117 (PMC3219374; doi:10.1186/cc10117)
Supplement: Additional file 1 — Supplementary tables and figures. Table S1: Number of funded ICU beds over the five-year study period. Table S2: Derivation of new diagnostic groupings from Acute Physiology and Chronic Health Evaluation (APACHE) III diagnostic codes. Table S3: Baseline characteristics and outcomes of study population and excluded group. Figure S1: Sensitivity analysis of cost saving of establishing a weaning unit for unit A. Figure S2: Sensitivity analysis of cost saving of establishing a weaning unit for unit D. [file cc10117-S1.PDF]

ADDITIONAL DATA FILE

Year of Study	Royal Infirmary of Edinburgh	St. John's Hospital, Livingston	Western General Hospital, Edinburgh	Total Number of Funded Level 3 Beds per Year
2002	11.75	4	8	23.75
2003	11.75	4	8	23.75
2004	12.25	4	8.25	25.25
2005	13	3.2	9	25.2
2006	13	3.2	9	25.2
Mean Annual Number of Funded ICU Beds	12.3	3.7	8.4	24.4

Table A1: Number of funded ICU beds over the five year study period. The years that have fractions of funded beds reflect an increase in bed numbers for a proportion of the year (usually over winter months).

New Diagnostic Grouping	APACHE III Diagnostic Code	n
Pneumonia - any cause	Pneumonia – aspiration/toxic<br>Pneumonia – bacterial<br>Pneumonia – fungal	89<br>649<br>4
Septic shock - any source	Septic shock – gastrointestinal tract<br>Septic shock – lungs<br>Septic shock – urinary tract infection<br>Septic shock – unknown origin	133<br>62<br>53<br>189
Trauma	Trauma – abdomen<br>Trauma – chest<br>Trauma – extremities<br>Trauma – face<br>Trauma – head/brain<br>Trauma – multiple sites without head/brain<br>Trauma – multiple sites plus head/brain<br>Trauma – pelvis<br>Trauma – spine	28<br>15<br>71<br>18<br>173<br>89<br>69<br>10<br>15

Table A2. Derivation of new diagnostic groupings from APACHE III diagnostic codes. APACHE= Acute Physiology and Chronic Health Evaluation.

	N missing	Study Cohort (n=7848)	Incomplete Episodes (n=208)	P value
Age Mean (SD)	None	57.0 (18.0)	55.9 (18.1)	0.39[a]
Sex n (% Female)	None	3366 (42.9)	88 (42.3)	0.87
APACHE II Score Mean (SD)	882/11	18.9 (8.2)	18.5 (7.2)	0.53[a]
CPR in 24 hours before ICU admission n (%)	None	686 (8.7)	9 (4.3)	0.03
Non-surgical Admission Diagnosis n (%)	38/112	5103 (65.3)	88 (91.7)	<0.001
Number of Co-morbidities n (%)	280/6			
None		5593 (73.9)	157 (77.7)	0.14[b]
1		1261 (16.7)	32 (15.8)	
2 or more		714 (9.4)	13 (6.4)	
Length of ICU stay (days)	None			
Mean (SD)		5.3 (9.0)	9.7 (13.6)	<0.001[b]
Median (IQR)		1.9 (0.9 to 5.7)	5 (1 to 13)	
Number of days ventilated	None			
Mean (SD)		4.2 (8.0)	8.8 (12.6)	<0.001[b]
Median (IQR)		1 (0 to 4)	4 (1 to 12)	
Mortality at ICU discharge n (%)	None	1830 (23.3)	42 (20.2)	0.29
Mortality at final hospital discharge n (%)	432/12	2558 (34.5)	73 (37.2)	0.45
Requiring Prolonged Mechanical Ventilation n (%)	None	349 (4.4)	23 (11.1)	<0.001

Table A3: Baseline characteristics and outcomes of study population and excluded group. P-values are reported for a comparison between the study cohort and incomplete transfers. The under 16 group (n=49) has not been described. SD= standard deviation; IQR=interquartile range; CPR=cardiopulmonary resuscitation; APACHE=Acute Physiology and Chronic Health Evaluation. [a]T-test; [b]Mann-Whitney U test.

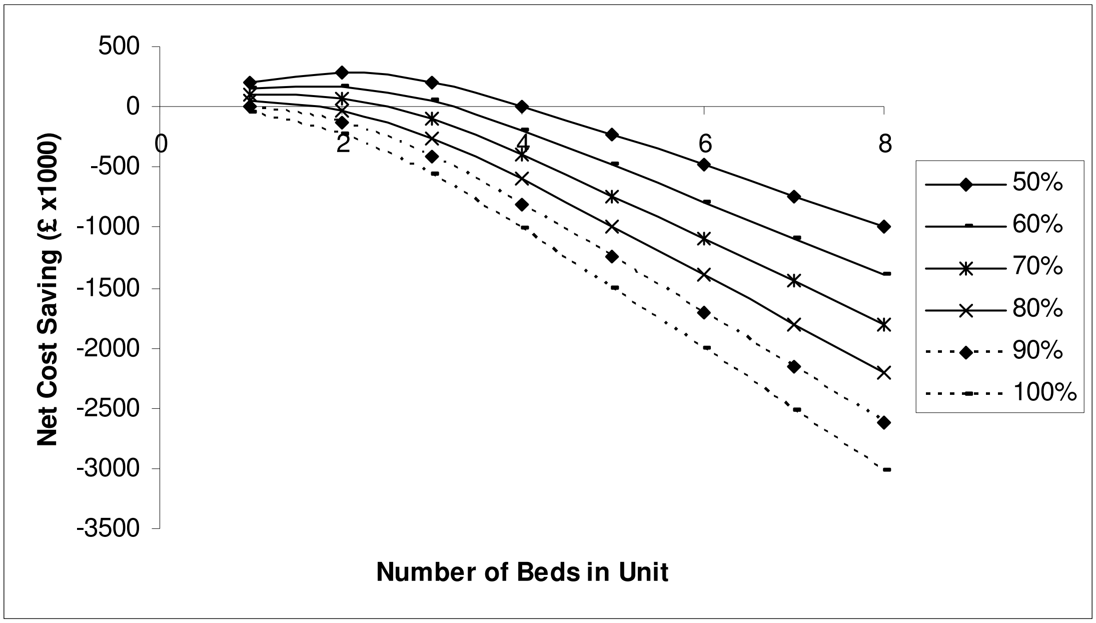

Figure A1: Sensitivity analysis of cost saving of establishing a weaning unit for Unit A. The cost of a weaning unit bed was varied from 50% of the cost of an ICU bed (top line) to 100% (bottom line). The sensitivity analysis was undertaken not taking account of unstable patients being transferred out of a weaning unit back to the ICU. A 3-bed unit with eligibility criteria for Unit A yields a net cost saving if the cost of a weaning unit bed is increased to 60% of the cost of an ICU bed, but not if it is increased to 70% or more.

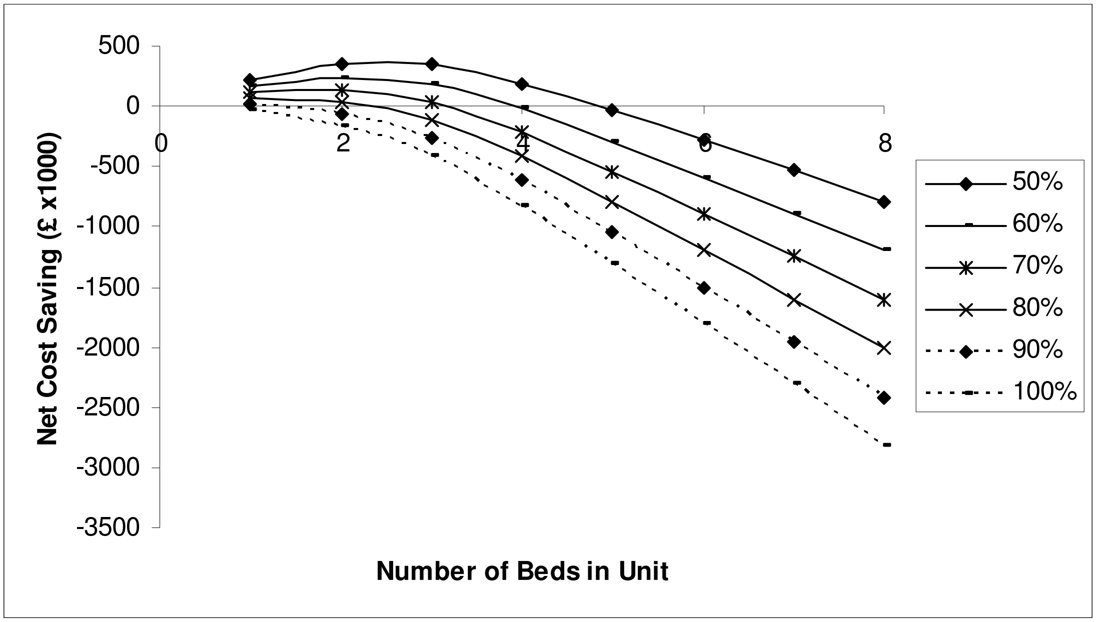

Figure A2: Sensitivity analysis of cost saving of establishing a weaning unit for Unit D. A 3-bed unit with eligibility criteria for Unit D yields a net cost saving if the cost of a weaning unit bed is increased to 70% of the cost of an ICU bed, but not if it is increased to 80% or more.
